# Dry Friction Properties of Diamond-Coated Silicon Carbide

**DOI:** 10.3390/ma16103640

**Published:** 2023-05-10

**Authors:** Yuefeng Du, Fangmin Xie, Jian Wang, Bin Xu, Huanyi Chen, Bineng Yan, Yanjiao Wu, Weifeng Huang, He Li

**Affiliations:** 1 Key Laboratory of Marine Materials and Related Technologies, Zhejiang Key Laboratory of Marine Materials and Protective Technologies, Ningbo Institute of Materials Technology and Engineering (NIMTE), Chinese Academy of Sciences, Ningbo 315201, China; 2 Ningbo FLK Technology Co., Ltd., Ningbo 315104, China; 3 State Key Laboratory of Tribology, Tsinghua University, Beijing 100084, China; 4 Center of Materials Science and Optoelectronics Engineering, University of Chinese Academy of Sciences, Beijing 100049, China

**Keywords:** mechanical seals, nano-diamond coatings, dry friction, silicon carbide

## Abstract

Dry friction between seal faces, caused by unstable or extreme operating conditions, significantly affects the running stability and service life of mechanical seals. Therefore, in this work, nanocrystalline diamond (NCD) coatings were prepared on the surface of silicon carbide (SiC) seal rings by hot filament chemical vapor deposition (HFCVD). The friction test results under dry environment reveals that the coefficient of friction (COF) of SiC–NCD seal pairs is about 0.07–0.09, which were reduced by 83–86% compared to SiC–SiC seal pairs. The wear rate of SiC–NCD seal pairs is relatively low, ranging from 1.13 × 10^−7^ mm^3^/N·m to 3.26 × 10^−7^ mm^3^/N·m under different test conditions, which is due to the fact that the NCD coatings prevent adhesive and abrasive wear between the SiC seal rings. The analysis and observation of the wear tracks illustrate that the excellent tribological performance of the SiC–NCD seal pairs is due to a self-lubricating amorphous layer formed on the worn surface. In conclusion, this work highlights a pathway to enable mechanical seals to satisfy the high application requirements under highly parametric working conditions.

## 1. Introduction

Mechanical seals, as important key components in rotating equipment to prevent leakage of process media or lubricating oil, ensure safe production, save energy and contain pollution, are widely used in major equipment in petrochemical, coal chemical, marine engineering, aerospace and other industries [1]. With the development of industry, energy and environmental requirements, the operating conditions of mechanical seal become more and more complex, involving such parameters as ultra-high pressure, ultra-high speed and high temperature [2,3]. Under normal circumstances, there is a uniform film of liquid between the rotating and static seal rings as a lubricating medium, preventing direct contact between the rings. However, deformation, overheating, vibration and wear of the seal face, resulting from high parameter condition, inevitably lead to direct contact and dry friction between the seal faces [3,4]. This situation leads to severe adhesive wear between seal end faces, resulting in excessive wear, shortened service life and even seal failure [5]. Therefore, improving the dry friction performance of seal end faces is an important way to increase the reliability of the mechanical seal under highly parametric working conditions.

Chemical vapor deposition (CVD) diamond films are widely used in the fields of wear-resistant devices such as coated tools, drawing dies, bearing carriers and nozzles [6,7,8,9,10] due to their excellent properties such as high hardness, low friction coefficient, high wear resistance and high thermal conductivity. Therefore, the fabrication of a diamond film on the end face of a mechanical seal ring can take full advantage of its unique mechanical and tribological properties and greatly improve the dry friction performance of mechanical seal. In the 1990s, Hollman et al., at Uppsala University [11], first deposited microcrystalline diamond (MCD) coatings on the surface of SiC seal rings using CVD techniques. The results showed that MCD can greatly reduce the friction force and wear rate of seal rings, and diamond coatings have great potential for application in mechanical seals. However, due to the rough surface of the MCD films, additional polishing steps are required, resulting in high production and time costs. This situation limits its further application on a commercial scale as antiwear coatings. Schade et al. investigated the tribological properties of four types of diamond coatings with different grain sizes [12]. The results showed the fine-grained diamond coatings have the lowest coefficient of friction (COF) and wear rate. The Argonne National Laboratory pioneered the production of ultra nanocrystalline diamond (UNCD) coatings [13] and successfully applied them to the mechanical seal of a multipurpose mechanical pump. They found that there is no obvious wear on the UNCD surfaces after 10 days of operation tests. Meanwhile, other researchers investigated the tribological properties of nanocrystalline diamond (NCD) [14,15,16,17,18,19,20]. Those results demonstrated the superior tribological performances of NCD and greatly increased the commercial application of diamond coatings in mechanical seals. Silva et al. [21,22,23,24,25,26,27] has systematically investigated the NCD growth mechanisms on the surface of silicon nitride (Si_3_N_4_) seal rings and explored the antiwear mechanisms of NCD-Si_3_N_4_ seal pairs under water conditions. The results show that the COF of NCD is 0.02–0.03 after the running-in regime and the limit normal load of wear tests depends on the surface roughness of substrate. Although diamond-coated mechanical seals have made great progress after years of development and research [28,29,30], there is little research on the dry tribological properties.

Therefore, in this paper, NCD with excellent tribological properties is applied to the SiC–SiC mechanical seal pairs. The dry friction and wear performances of NCD–SiC seal pairs are investigated, with the aim of effectively improving the service life and reliability of highly parametric mechanical seals under extreme working conditions.

## 2. Materials and Methods

### 2.1. Preparation of Diamond Coatings

**Figure 1:**
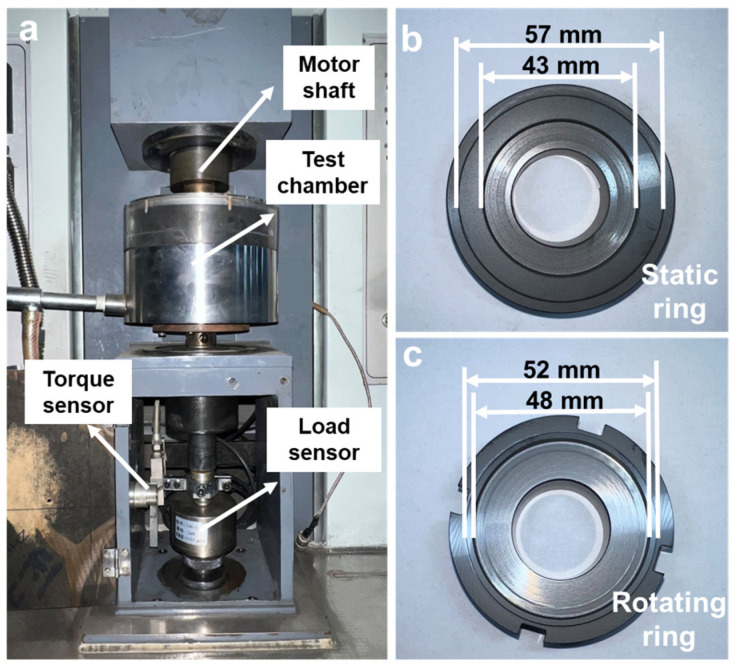
(**a**) Photographs of testing machine, (**b**) Static seal ring and (**c**) Rotating seal ring.

In this paper, the NCD coatings were produced by HFCVD. The rotating and static seal rings were porous carbonized silicon carbide (p-GSiC) produced by NingBo FLK Technology Co., Ltd. (Ningbo, China). As shown in Figure 1b,c, the outer and inner diameters of rotating seal rings were 52 mm and 48 mm. The sealing surface of the static sealing ring with an outer diameter of 57 mm and an inner diameter of 43 mm was deposited with nano-diamond. Before depositing, the seal surfaces of static seal rings were ground with diamond powder with a particle size of 30 μm for 5 min until the Ra value was 0.3 μm, aiming to improve the nucleation density of diamond. Then, the static seal rings were sonicated by alcohol and acetone for 5 min.

After drying with nitrogen, the rotating seal ring was placed in the deposition chamber. Four evenly distributed tantalum filaments were fixed at a distance of 10 mm above the surface of the sealing ring. The deposition process is divided into two steps: carbonization of the tantalum filaments and deposition of the nano-diamond coating. The ratio of H_2_/CH_4_ was 400/20. The gas pressure was 2 kPa. The power gradually increased to 4400 W, and the duration was 20 min. The ratio of H_2_/CH_4_/N_2_ was 400/20/60. The gas pressure was 2.5 kPa. The deposition time was 18 h.

### 2.2. Dry Tribological Test

The dry tribological performance tests of NCD were conducted on a high-speed end-face friction and wear testing machine at room temperature, as shown in Figure 1a, the details of this set up were shown in our former work [28]. As shown in Table 1, dry tribological tests were divided into two groups: Group 1: dry tribological performance was tested at 500, 1000, 1500 and 2000 rpm rotational speed at fixed normal load of 100 N; Group 2: the dry tribological performance was tested at 100, 150, 200 and 250 N normal load at a fixed rotational speed of 1000 rpm. All tests were terminated when the COF suddenly increased, indicating lubrication failure. In order to reduce errors, each test was performed at least three times.

### 2.3. Characterizations

The surface and cross-sectional morphology of the nano-diamond coating were observed by scanning electron microscope (SEM, Quanta FEG250, FEI, Hillsboro, OR, USA). The Raman spectra of the coatings were characterized by Raman system (Raman, LabRAM, Odyssey, HORIBA FRANCE SAS, Kyoto, Japan).

After the tribological tests, the morphologies of the wear tracks were observed by optical microscopy (Leica DM2500 M, Leica, Wetzlar, Hessian, Germany) and SEM. The Raman spectra of the wear tracks were measured by LabRAM Raman system. The high-resolution cross-sectional morphologies of the surface layer of the wear tracks were investigated using high-resolution transmission electron microscopy (HRTEM, Talos F200X, Thermo Fisher Scientific, Waltham, MA, USA). The TEM samples were prepared using focused ion beam microscopy (FIB, Helios 5 CX, Thermo Fisher Scientific, Waltham, MA, USA).

## 3. Results

### 3.1. NCD Characterization

**Figure 2:**
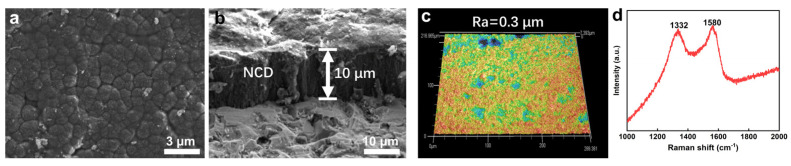
(**a**) Top view, (**b**) Cross-sectional view, (**c**) 3D surface profile and (**d**) Raman spectrum of NCD coatings.

After the NCD was prepared on the surface of the SiC sealing rings, the surface morphology and cross-section of the NCD were observed and analyzed, as shown in Figure 2. From the top view of the NCD in Figure 2a, it can be seen that the surface of the NCD consists of many small diamond particles. Figure 2b shows the cross-sectional view of the NCD coating. It can be seen that the thickness of the NCD reaches about 10 μm after 15 h of growth. Figure 2c shows the three-dimensional surface profile of the NCD coating measured by confocal laser scanning confocal microscope. The results in this figure show that the surface of the NCD is relatively flat. Meanwhile, the surface roughness was about 0.3 μm, which is consistent with the roughness of the seal ring after grinding in Section 2.1, indicating that the preparation of the NCD does not significantly affect the surface roughness of the seal ring. Figure 2d shows the Raman spectrum of NCD, in which two obvious characteristic peaks at 1332 cm^−1^ and 1580 cm^−1^ can be seen, which are related to the typical Raman spectrum of nano-diamond [31,32,33,34]. Among them, 1332 cm^−1^ is a typical diamond characteristic peak [35,36]. During the growth and preparation process of NCD, some graphite phases are inevitably formed at the grain boundaries, and the graphite signal is more sensitive in the Raman line, so there is a characteristic peak at 1580 cm^−1^ characteristic peak [37].

### 3.2. Friction Testing Results

**Figure 3:**
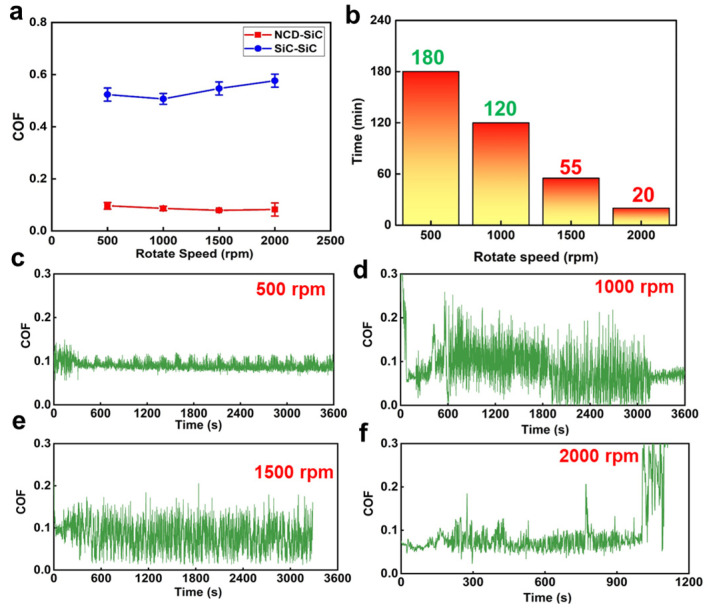
(**a**) COF of SiC–SiC sealing pairs and SiC–NCD sealing pairs at different rotating speeds under the fixed normal load of 100 N; (**b**) operating life of the NCD–SiC sealing pairs under different speeds; COF of NCD–SiC pairs as a function of times under (**c**) 500 rpm, (**d**) 1000 rpm, (**e**) 1500 rpm and (**f**) 2000 rpm.

**Figure 4:**
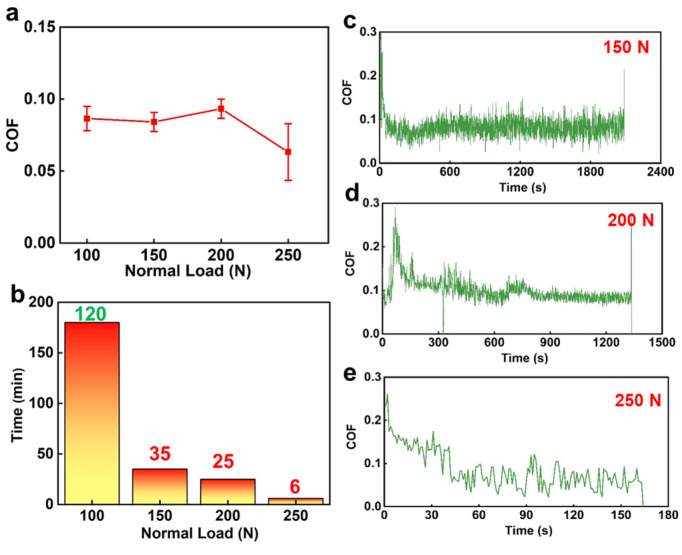
(**a**) COF and (**b**) operating life of SiC–NCD sealing pairs at different normal loads under the fixed rotating speed of 1000 rpm; COF of NCD–SiC pairs as a function of times under (**c**) 150 N, (**b**) 200 N and (**e**) 250 N.

After the dry friction tests, the test results were statistically evaluated and analyzed. The results are shown in Figure 3 and Figure 4. Figure 3a shows the results of the dry friction tests of the SiC–SiC seal pairs and the SiC–NCD seal pairs at different rotational speeds under 100 N fixed normal load. The blue line in Figure 3a represents the COF curve of the SiC–SiC seal pairs, changing with rotational speeds. The COF of this pair first decreased and then increased as the speed increased. However, the overall change was not significant and remains at 0.5–0.6. In addition, the tests were accompanied by strong vibrations and noise. The red line in the figure is the COF curve of SiC–NCD seal pairs, changing with the rotational speeds. Compared to the SiC–SiC seal pairs, the COF of the NCD–SiC pairs has decreased significantly, gradually decreasing from 0.09 to 0.07 as the rotation speed increases. Figure 3b shows the service life of the NCD–SiC seal pairs at different rotation speeds under dry friction. Taking 180 min as the upper limit of the friction tests, this pair could meet the service life requirements at 500 and 1000 rpm with stable running, and without any obvious vibration and noise. At 1500 rpm, the COF increased sharply at the test duration of 55 min. Meanwhile, obvious vibrations and noises occurred, which is considered an operation failure. At this point, it can be concluded that 55 min is the service life of the NCD–SiC pairs under 1500 rpm. At 2000 rpm rotational speed, the service life was shortest and is 20 min. Figure 3c–f shows the COF of the NCD–SiC pairs as a function of time for different rotational speeds. Except for the relatively stable curve at 500 rpm, the COF varied greatly, but they all fluctuated around 0.07–0.09. These results show that the application of NCD can significantly improve the lubrication condition of SiC–SiC seal pairs under dry friction.

Based on the work in Figure 3, the dry friction performances of the SiC–NCD seal pairs were tested under different loads at 1000 rpm fixed speed, and the results are shown in Figure 4. From Figure 4a, it can be seen that the COF suddenly drops to 0.06 under the normal load of 250 N and also fluctuates around 0.08 under other load conditions, indicating that the influence of load and speed on the COF is not significant. However, it can be seen from Figure 4b that the load has a great influence on the service life. When the load is increased to 150 N, the life of the pair dropped drastically to 35 min. When the load is increased to 250 N, the wear life can be maintained for only 6 min. Figure 4c,d shows the variation of COF with time under different normal loads.

### 3.3. Wear Rate

**Figure 5:**
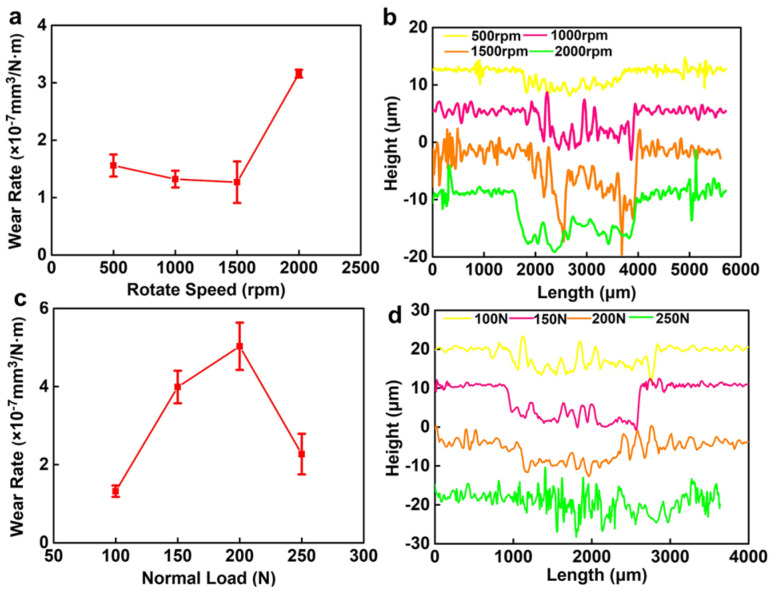
(**a**) Wear rate of NCD coatings and (**b**) cross-section profiles of each wear track under different rotating speeds; (**c**) wear rate of NCD coatings and (**d**) cross-section profiles of each wear track under different normal loads.

After the dry friction tests, the cross-sectional profiles of the wear tracks were scanned and characterized using laser confocal microscopes and the wear rate of the NCD coating was calculated under different test conditions. Figure 5a shows the wear rate of the NCD coating as a function of the rotational speed at 100 N fixed normal load. It can be seen that when the rotational speed increased from 500 rpm to 1500 rpm, the wear rate decreases from 1.52 × 10^−7^ mm^3^/N·m to 1.13 × 10^−7^ mm^3^/N·m. However, when the speed increased to 2000 rpm, the wear rate suddenly increased to 3.26 × 10^−7^ mm^3^/N·m. Figure 5b shows the cross-sectional profiles of each wear track on the NCD surface at different rotational speeds. It can be concluded that as the rotational speed increases, the cross-sectional profiles of the wear tracks gradually become distinct. The width gradually increased from 1800 μm to 2200 μm, and the depth gradually increased to 10 μm, reaching the thickness of the NCD and indicating that the NCD is fully worn out at this time. Figure 5c is a high magnification SEM image of the NCD wear rate as a function of normal load at 1000 rpm fixed rotational speed. In contrast to Figure 4a, when the load was increased from 100 N to 150 N, the NCD wear rate increased from 1.32 × 10^−7^ mm^3^/N·m to 2.65 × 10^−7^ mm^3^/N·m. Then, as the load increased to 200 N, the wear rate showed a downward trend and dropped to 2.51 × 10^−7^ mm^3^/N·m. However, when the load increased to 250 N, the wear rate dropped drastically to 0.98 × 10^−7^ mm^3^/N·m. Figure 5d shows the cross-sectional profiles of each wear tracks of NCD at different normal loads. At 150 N normal load, the width of the wear track did not change significantly, but the depth increased from about 6 μm to 8–9 μm, but less than 10 μm, which is the depth of the NCD. As the load increased, the depth and width of the wear track started to decrease. When the load increased to 250 N, the profile of the wear track became less distinct. In general, the change of the wear track corresponds to the change in the wear rate. However, it is also explained that the cause of the operational failure of the sealing pairs under higher load is not due to the wear failure of the NCD.

### 3.4. Wear Tracks Morphology

In order to study the lubrication mechanism of NCD, the wear tracks were analyzed and characterized.

#### 3.4.1. SiC–SiC Sealing Pairs

**Figure 6:**
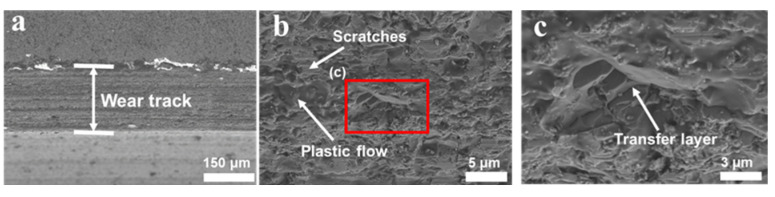
(**a**) SEM image of wear tracks on SiC surface; (**b**) high-magnification SEM images of wear tracks; (**c**) high-magnification SEM images of red box in the subfigure (**b**).

First, the wear tracks of the SiC–SiC seal pair were analyzed using SEM, as shown in Figure 6. There were a large number of scratches caused by abrasive wear on the surface of the wear tracks. At the same time, the plastic flow caused by the temperature rise of the interface during the friction process was observed in Figure 6a,b. In addition, there were a large number of transfer layers caused by adhesive wear on the surface of the wear track, as shown in Figure 6c. According to the analysis of the wear track, the reasons for the high COF and the strong vibrations between SiC–SiC seal pairs are the severe adhesive wear during dry friction.

#### 3.4.2. SiC–NCD Sealing Pairs

**Figure 7:**
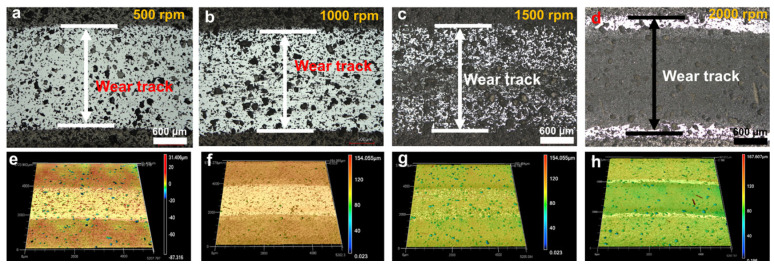
(**a**–**d**) Optical images and (**e**–**h**) 3D surface profiles of wear tracks under different rotating speeds.

**Figure 8:**
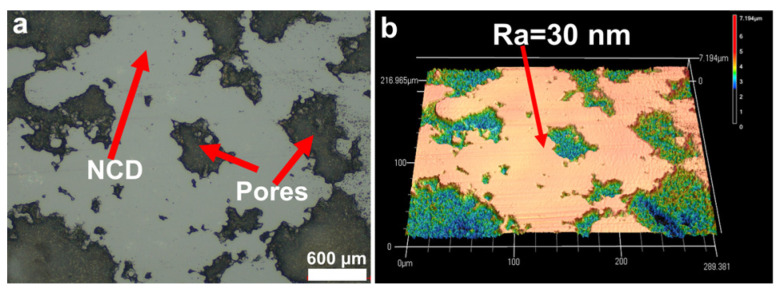
(**a**) High-magnification optical image and (**b**) surface profile of wear tracks.

**Figure 9:**
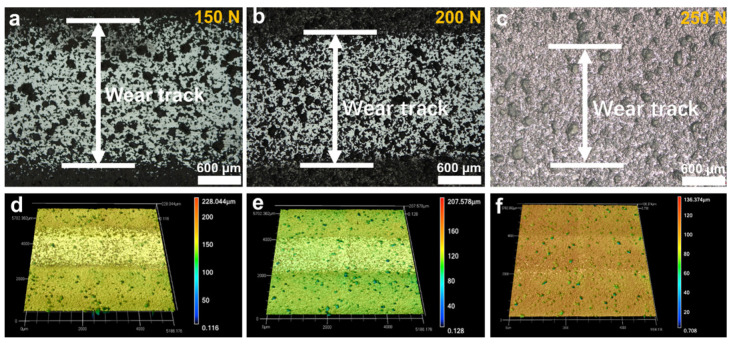
(**a**–**c**) Optical images and (**d**–**f**) 3D surface profiles of wear tracks under different normal loads.

Optical microscope and laser confocal microscope were then used to observe and analyze the morphology of the wear marks. The results are shown in Figure 7, Figure 8 and Figure 9. Figure 7 shows the optical photographs and 3D profiles of the wear tracks at different rotational speeds. Dense black and bright areas distributed in the wear tracks surface. Figure 8 is the higher magnification view of the wear tracks. It can be seen that the black area is caused by the internal pores of the p-GSiC matrix. The surface was smooth with no obvious scratches. The surface roughness of bright areas was only 30 nm. As the rotational speed increased, the area of the bright areas gradually decreased. When the rotational speed increased to 1500 rpm, the area of the bright areas decreased significantly. Until the rotational speed increased to 2000 rpm, there was no longer any bright area in the wear track. This result, combined with the contour line of the wear track in Figure 5b, also shows that the NCD in the wear track is completely worn out at this time.

Figure 9 is the optical image and the 3D profile of the wear track under different loads. It is clear from the image that the width of the wear track significantly increases at 150 N compared to the wear track at 100 N in Figure 7b, but the width of the wear track significantly was reduced at 200 N. Although the service life was shortest at 250 N, the photo of the wear track in Figure 9c shows that there is no obvious wear track on the surface of the seal ring, indicating that NCD does not cause obvious wear. These results are also consistent with the cross-sectional profiles of the wear track in Figure 5d.

**Figure 10:**
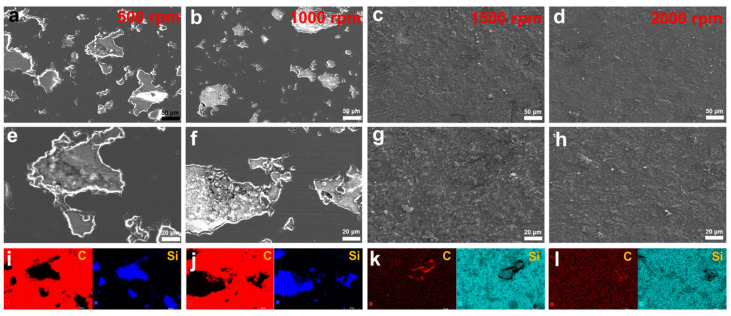
(**a**–**d**) SEM images, (**e**–**h**) high-magnification SEM images and (**i**–**l**) EDS mapping diagrams of wear tracks under different rotating speeds.

**Figure 11:**
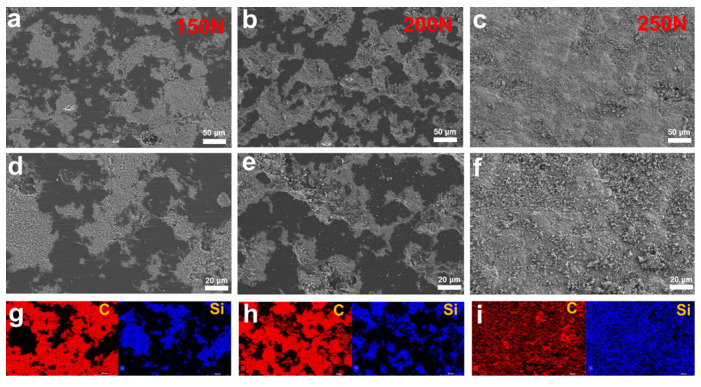
(**a**–**c**) SEM images, (**d**–**f**) high-magnification SEM images and (**g**–**i**) EDS mapping diagrams of wear tracks under different rotating speeds.

In order to further analyze the wear mechanism of NCD–SiC seal pairs under different working conditions, SEM was used to analyze and observe the wear tracks. The results were shown in Figure 10 and Figure 11. Figure 10 shows the SEM images and the energy spectrum mapping images of the wear tracks under different rotational speed conditions. The SEM photographs of the wear tracks at the rotational speed of 500 rpm and 1000 rpm were shown in Figure 10a,b,e,f. The pits in the wear tracks came from porous structures in the matrix. After 180 min of friction test, the surface outside the pits were very flat and smooth, with no visible scratches. This area was the bright area in Figure 8 and Figure 9. At the same time, the energy dispersive spectrometer (EDS) mapping diagrams of wear tracks were illustrated in Figure 10i,j; it can be seen that the element in the smooth area is C, which indicates that the smooth area is NCD and that NCD is not completely worn out at this time. When the rotational speed increased to 1500 rpm and 2000 rpm, as shown in Figure 10c,d,g,h, it can be seen that there is no smooth region in the wear track with some wear debris attached to the wear track. Combined with the diagrams of the wear track EDS in Figure 10k,l, it can be concluded that there is no obvious C-element signal in the wear track, which means that the NCD is almost worn out. At the same time, the optical characterization results in Figure 7 revealed that the presence of NCD avoids the serious adhesion wear caused by the direct contact of SiC ceramic and greatly enhances the wear life of seal pairs.

Figure 11 shows the SEM images and energy spectrum mapping images of wear tracks under different loads. Similar to Figure 10, smooth NCD can be seen in the wear tracks. However, the difference is that the pores of the wear tracks are filled with a certain amount of wear debris. From the mapping results in Figure 11g–i, it can be seen that the wear debris element is mainly silicon, which indicates that the wear debris comes from the rotating seal ring. The amount of wear debris increased significantly with the increasing normal load. In particular, at a normal load of 250 N, the wear debris almost covered the entire surface of the wear track. This phenomenon shows that, as the normal load increased, the mechanical limit of the rotating seal ring matrix was reached, resulting in heavy wear and damage. A large amount of wear debris was accumulated in the wear tracks, which destroys the stable operating condition of the mating pair, resulting in a high COF, obvious vibration and noise. This phenomenon is also another important reason for the wear failure of the seal pair.

## 4. Discussion

**Figure 12:**
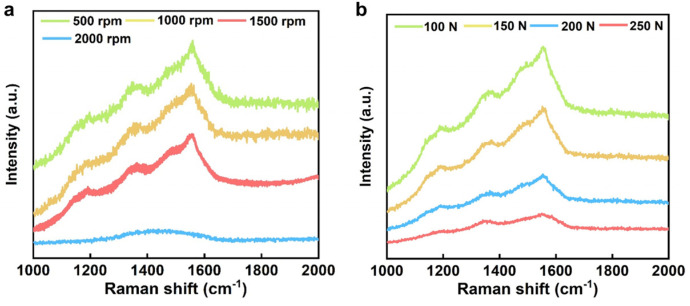
Raman spectrum of wear tracks of (**a**) different rotating speeds and (**b**) normal loads.

In order to explore the wear mechanism, Raman spectroscopy was used to characterize the surface of the wear tracks, as shown in Figure 12. Figure 12a,b presents the Raman spectra of the NCD wear tracks under different rotational speeds and different normal loads, respectively. The difference from the original Raman peak in Figure 2d is that the G peak at 1580 cm^−1^ is significantly enhanced, indicating that the NCD surface undergoes graphitization under the action of friction. However, when the rotation speed increased to 2000 rpm, the Raman spectrum of the NCD was flattened and broadened with no obvious characteristic peaks, indicating that the NCD is highly worn and completely amorphized. In Figure 12b, although the NCD was tested for a short time under different loading conditions, the phenomenon of G-peak enhancement was also observed.

**Figure 13:**
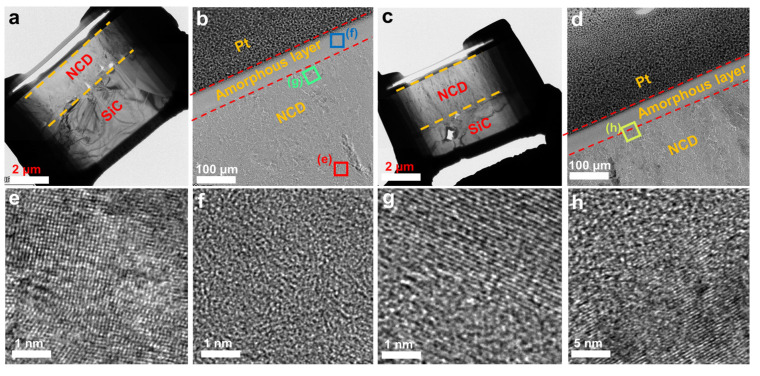
(**a**) SEM image of FIB specimen and (**b**) TEM image of subface of wear track under 100 N normal load; (**c**) SEM image of FIB specimen and (**d**) TEM image of subface of wear track under 250 N normal load; high-magnification TEM image of (**e**) NCD lattice structure, (**f**) amorphous layer and (**g**,**h**) graphitization area.

To better observe the graphitization and amorphous transformation of the NCD surface during the friction process, TEM was used to observe and characterize the subsurface of the wear tracks. Figure 13a,c presents the FIB specimens SEM images of the wear track under 100 N and 250 N normal load. The top layer of the sample is NCD, and the bottom layer is SiC substrate. Figure 13b,d presents the higher magnification TEM images of the blue square in Figure 13a,b. Between the Pt protective layer and the NCD layer was a layer with an average thickness of about 100 μm, which has significant structural differences from NCD. Figure 13e is a high-resolution image of the NCD layer of the red square in Figure 13b, where the lattice structure and atomic arrangement of diamond can be clearly seen. Figure 13f is a high-resolution image of the interlayer of the blue square in Figure 13b, showing that there is no crystal structure, which means that the interlayer is an amorphous layer formed by the amorphous transformation of NCD. Figure 13g,h presents the high resolution images of the region near the amorphous layer. Compared to Figure 13e, the lattice structure in this region has changed significantly and is now more inclined towards a graphitic layer structure, which is the transition stage before complete amorphization. At the same time, there were still small amorphized regions in Figure 13g, as shown by the yellow dotted line area in this figure. This phase transition phenomenon is consistent with the existing research results, which indicates that diamond may cause sliding-induced graphitization or rehybridization under the combined influence of friction temperature and stress [38,39,40]. So, it can be concluded that an amorphous layer with uniform thickness plays an important lubricating role as a self-lubricating film, which is formed in the initial stage of friction, and its existence can significantly reduce the COF of the mechanical seal partner in the state of dry friction.

## 5. Conclusions

In summary, this work deposited the NCD coatings on the surface of SiC seal rings by HFCVD. The tribological tests results show that the NCD coatings can significantly enhance the tribological properties of SiC–SiC seal pairs in dry environment. The main conclusions are as follows:

(1) NCD coatings with thickness of 10 μm and surface roughness of 0.3 μm were successfully deposited on the surface of SiC static seal rings. The COF of SiC–NCD seal pairs was about 0.07–0.09, which was reduced by 83–86% compared to SiC–SiC seal pairs. The wear rate of NCD–SiC ranged from 1.13 × 10^−7^ mm^3^/N·m to 3.26 × 10^−7^ mm^3^/N·m at various normal loads and speeds.
(2) The observations of wear tracks indicate that the presence of NCD coatings prevent serous adhesive wear and abrasive wear between SiC seal rings. The main reasons for limiting the service life of NCD–SiC seal pairs are the failure of the NCD coatings and the accumulation of wear debris.
(3) Wear tracks analysis shows that a self-lubricating amorphous layer was formed by the amorphous transformation of NCD during friction testing due to the sliding-induced graphitization or rehybridization of diamond. This phase transition phenomenon is the reason for the excellent tribological performances of NCD–SiC seal pairs.

## Figures and Tables

**Table 1:** Dry tribological performance test arrangements.

Number	Rotating Speed (rpm)	Normal Load (N)
Group 1	500, 1000, 1500 and 2000	100
Group 2	1000	100, 150, 200 and 250

## Data Availability

Not applicable.
